# pH Alteration in Plant-Mediated Green Synthesis and Its Resultant Impact on Antimicrobial Properties of Silver Nanoparticles (AgNPs)

**DOI:** 10.3390/antibiotics11111592

**Published:** 2022-11-10

**Authors:** Amalia Miranda, Tamara Akpobolokemi, Etelka Chung, Guogang Ren, Bahijja Tolulope Raimi-Abraham

**Affiliations:** 1 Institute of Pharmaceutical Science, School of Cancer and Pharmaceutical Sciences, King’s College London, Waterloo Campus, Franklin Wilkins Building, Stamford Street, London SE1 9NH, UK; 2 School of Physics, Engineering and Computer Science, University of Hertfordshire, Hatfield AL10 9AB, UK

**Keywords:** antimicrobial efficacy, green synthesis, pH, silver nanoparticle

## Abstract

Plant-mediated green synthesis is a cost-effective and eco-friendly process used to synthesize metallic nanoparticles. Experimental pH is of interest due to its ability to influence nanoparticle size and shape; however, little has been explored in comparison to the influence of this parameter on the therapeutic potential of resultant metallic nanoparticles. Our work investigated the influence of pH alternation on antimicrobial properties of plant-mediated green synthesized (using *Spinacia oleracea* leaf extract) silver nanoparticles. We further investigated if the antimicrobial activity was sustained at 8 weeks (after initial green synthesis). Antimicrobial properties were evaluated against *Escherichia coli*, *Staphylococcus aureus*, and *Candida albicans*. Our work confirmed that experimental pH in plant-mediated green synthesis of silver nanoparticles influenced their resultant antimicrobial properties. Silver nanoparticles generated at experimental pH 4.5, and nine showed activity against *E. coli* which was sustained at various levels over 8 weeks. No antimicrobial activity was observed against *S. aureus*, and weak antimicrobial activity against *C. albicans*. These interesting findings highlight the importance of experimental pH. Further understanding of the role experimental pH plays on resultant metallic nanoparticle properties as it relates to biological and therapeutic impact is required, which will have an impact on wider applications beyond antimicrobial activity.

## 1. Introduction

Green synthesis of inorganic nanoparticles has been explored in a broad range of therapeutic and non-therapeutic areas due to its advantages as a cost-effective, eco-friendly process with reduced generation of harmful by-products. Plants can synthesize metallic nanoparticles due to their ability to detoxify metals via a chelation mechanism when bioaccumulation occurs [1]. Organic compounds in plants include primary and secondary metabolites, which facilitate the reduction of metal ions and the resultant stabilization towards formation as nanoparticles [1,2]. However, it is important to note that the characteristic of the resultant plant-mediated green synthesized metallic nanoparticle properties is highly dependent on the part of the plant used due to varied phytochemical compositions [2]. For example, the work by Marchiol et al., 2014 investigated the in vivo formation of silver nanoparticles (AgNP) in living plants (namely *Brassica juncea*, *Festuca rubra* and *Medicago sativa*) which identified differences in AgNP formation (including the absence of) size and shape depending on plant [3]. Interestingly, the highest uptake and translocation of silver (Ag) was observed in plant leaves (in all species) [3]. *Spinacia oleracea* is a popular plant possessing an abundance of phytochemicals, and the whole plant has been successfully used to generate metallic nanoparticles [4,5,6]. The leaf extract, which we used in our work, has been used to a lesser extent in the green synthesis of metallic nanoparticles.

Experimental parameters such as pH, temperature, and reaction or incubation time influence the plant-mediated green synthesis of metallic nanoparticles [7]. Natural plant pH can vary in different areas of the plant and have an impact on plant extract antioxidant activity [8,9,10]. The experimental pH is of great interest as this has been shown to influence the size and shape of resultant metallic nanoparticles [11,12]. Morones et al. (2005) demonstrated the size-dependent bactericidal properties produced by AgNPs [13]. Another study also found that the small AgNPs (10 nm) were found to be cytotoxic for human lung cells, while no activity was found in the larger AgNPs size [14]. The difference in bioactivity is the result of the simultaneous mechanism when AgNPs-bacteria interaction occurs, including the diffusion of small AgNPs and Ag ions into the cell, disruption of the cell membrane, and the protein and DNA alteration within intracellular media [15]. However, little has been explored in comparison to the influence of experimental pH on the biological functionality and therapeutic potential of the resultant metallic particles.

In this study, our work aimed to investigate the influence of pH alternation on antimicrobial properties of plant-mediated green synthesized (using *Spinacia oleracea* leaf extract) AgNPs. We further investigated if any antimicrobial activity observed was sustained for 8 weeks (post initial green synthesis). Ag and, in turn, AgNPs are well known for their antimicrobial properties, including against drug-resistant pathogens. [16,17]. Antimicrobial properties were evaluated against *Escherichia coli*, *Staphylococcus aureus*, and *Candida albicans*. This selection was based on the World Health Organization (WHO) priority pathogens list for new antibiotics, where *E. coli* is on the critical priority list in the Gram-negative group while *S. aureus* is on the high priority list in the Gram-positive group [18]. In addition to antimicrobial properties, we investigated the shape and size difference of resultant AgNPs after pH alternation. It is hypothesized that nanoparticle shape and size influence uptake as well as an antimicrobial effect [19,20]. Studies have shown that via plant-mediated green synthesis, larger nanoparticles are formed at acid pH (2–4) compared to basic pH [21]. Whilst at basic pHs, metallic nanoparticles generated have shown to be smaller in size and produced a higher yield [12]. The pH alternation range used in this study was pH 5–9. 

## 2. Results

### 2.1. Sample Preparation

#### 2.1.1. Visual Observation

**Figure 1:**
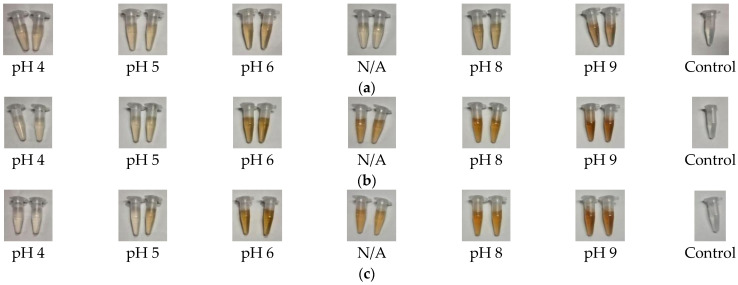
The visual observation of the reaction solution affected by pH was observed after (**a**) 0 h, (**b**) 2 h, and (**c**) 3 h of synthesis [N/A refers to sample without pH adjustment].

It has been well established from various studies that the Surface Plasmon Resonance (SPR) band is an important characteristic of metallic nanoparticles responsible for absorbing light [22,23]. This band is located in the blue light region (390–450 nm), causing the reflection of green and red light when placed on a white background [24]. As a result, a yellow colour will be reflected. This phenomenon offers a visual indication of the formation of AgNPs. Figure 1 depicts the visual investigation of the reaction solution affected by pH over the hours of synthesis. 

As shown in Figure 1, the samples prepared in this study formed a specific perceived colour, ranging from light yellow to reddish-brown. Sample 9 formed the most intense reddish-brown colour, followed by samples 6 and 8. Meanwhile, a light-yellow colour is reflected by AgNP-4, AgNP-5 and AgNP-N/A. The colour of those samples became more intense as the increasing hours of synthesis for AgNP-6, AgNP-N/A, AgNP-8, and AgNP-9. In contrast, a reduction of colour intensity appeared in AgNP-4 and AgNP-5. At the end of synthesis (3 h), reddish-brown colour was observed in AgNP-6, AgNP-N/A, AgNP-8, and AgNP-9, while AgNP-4 and AgNP-5 transformed appeared an almost clear solution similar to the control.

#### 2.1.2. UV-Vis Spectroscopy Analysis

**Figure 2:**
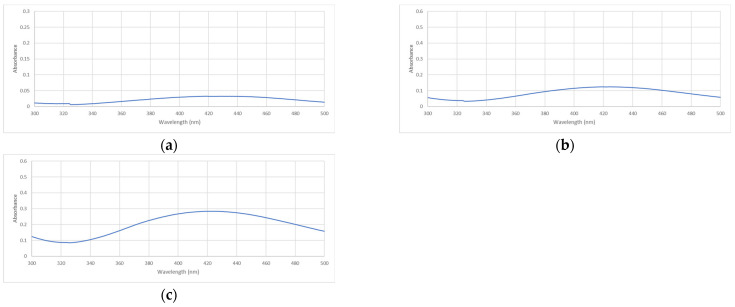
UV-Vis spectra of samples observed after 3 h of synthesis affected by pH: (**a**) AgNP-6, (**b**) AgNP-8, and (**c**) AgNP-9.

SPR band is produced by the electromagnetic field that induces collective oscillation of the conduction band at the surface of the nanoparticle [22,23]. Since the SPR band was found to be the unique characteristic of metallic nanoparticles, UV-Vis analysis became an effective and reliable characterization technique for evaluating the synthesis and stability of AgNPs [25]. The number of peaks, time of occurrence, and peak shifting of the SPR band provide useful information about the properties of nanoparticles. It was known that the single SPR band corresponds to the spherical nanoparticle, while more than 1 SPR band may indicate the anisotropic particle [26]. Additionally, silver nanoparticle properties such as size and shape vary as well as molarity and concentration from the UV-Vis spectroscopy peak value [27]. For example, a shift of the SPR peak to a longer wavelength (red-shift) can indicate smaller particle size formation [28]. UV-Vis spectra showed a strong absorption band was detected at the wavelength around 390–450 nm from UV-Vis spectra of samples AgNP-6, AgNP-8, and AgNP-9, while there was no SPR band observed for AgNP-4, AgNP-5, and AgNP-N/A after three hours of synthesis (Figure 2) which was in not supported by visible observation studies which show a distinct colour change. The difference observed are likely due to low absorbance values (maximum wavelength (*λ*max) and absorbance of the SPR band detected are presented in Table 1). 

### 2.2. Nanoparticle Characterization

#### 2.2.1. Dynamic Light Scattering (DLS) Analysis

DLS is an instrument to measure the hydrodynamic size of particles by analyzing the modulation of the scattered light intensity as a function of time [29]. This current study used DLS to obtain Z-average (d.nm) and Polydispersity Index (pDI) values, which are important parameters in nanoparticle characterization [30,31]. Table 2 provides the average particle size and the pDI value for AgNPs prepared at various pHs. 

Size changes of the particles were observed as the pH increased. The largest particle size was achieved with AgNP-4 and began to decrease as the pH increased up to 6. The sample without pH adjustment (AgNP-N/A) had a greater particle size compared to pH 6. The sample with the smallest particle size was AgNP-8 at 91.75 nm, and pH elevation to 9 resulted in a size increase to 263.6 nm. The particle size distribution followed the same pattern where the largest pDI value was found in AgNP-4 and the smallest in AgNP-8. A non-parametric analysis using the Kruskal-Wallis test demonstrated that the pH used in this study did not significantly affect the particle size, H(5) = 5.906, *p* = 0.315.

#### 2.2.2. Fourier Transform Infrared Spectroscopy

**Figure 3:**
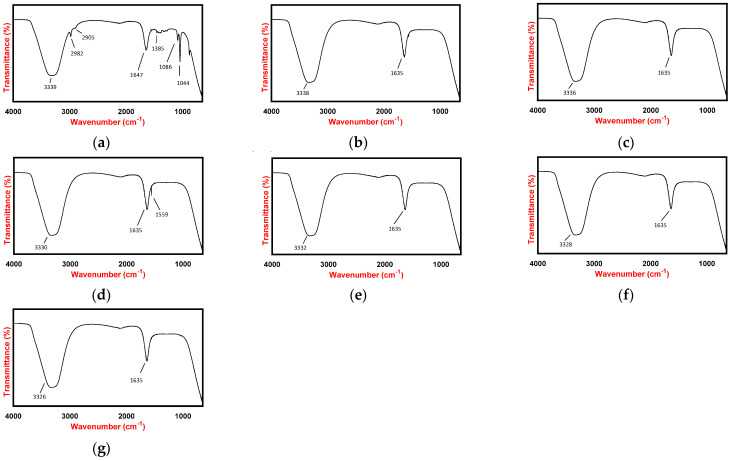
FTIR spectra of samples after 3 h of synthesis affected by pH: (**a**) *Spinacia oleracea* leaf extract, (**b**) AgNP −4, (**c**) AgNP−5, (**d**) AgNP−6, (**e**) AgNP−N/A, (**f**) AgNP−8, and (**g**) AgNP−9.

FTIR spectra of *Spinacia oleracea* leaf extract and AgNP sample prepared in various pH are provided in Figure 3. The FTIR spectra of *Spinacia oleracea* leaf extract depicts some strong peaks at 3339 cm^−1^, 1647 cm^−1^, and 1044 cm^−1^, which corresponds to the O-H stretching, C=O stretching, and C-O stretching, respectively. Besides, some weak signals appeared at wavenumber 2982 cm^−1^ and 2905 cm^−1^ referring to C-H stretching and 1385 cm^−1^ to C-H bending of the aliphatic group. The medium signal at 1086 cm^−1^ could be attributed to C-N stretching from the tetrapyrrole ring of chlorophyll or flavonoid. The similar FTIR spectra pattern of AgNPs was previously described by Kumar et al., 2017, and Gariboet et al., 2020 [32,33]. AgNPs prepared in all pH showed a strong peak at a wavelength around 3332–3338 cm^−1^ and 1635 cm^−1^, referring to O-H stretching and C=O stretching. 

#### 2.2.3. Nanoparticle Morphology

**Figure 4:**
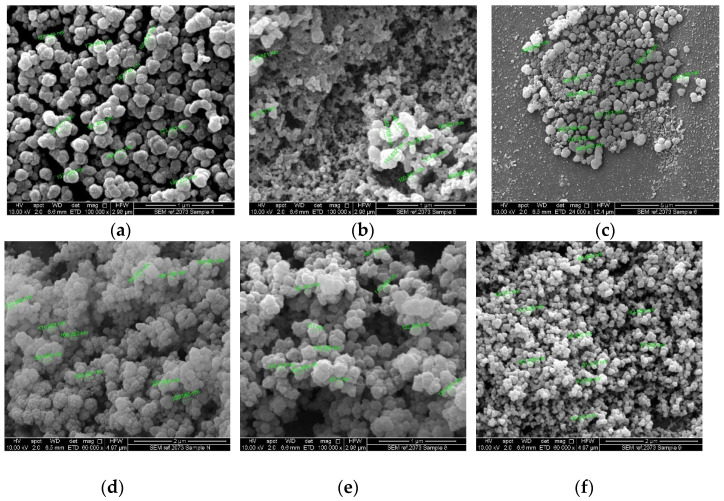
SEM images samples (**a**) AgNP-4; (**b**) AgNP-5; (**c**) AgNP-6; (**d**) AgNP-N/A; (**e**) AgNP-8; (**f**) AgNP-9.

**Figure 5:**
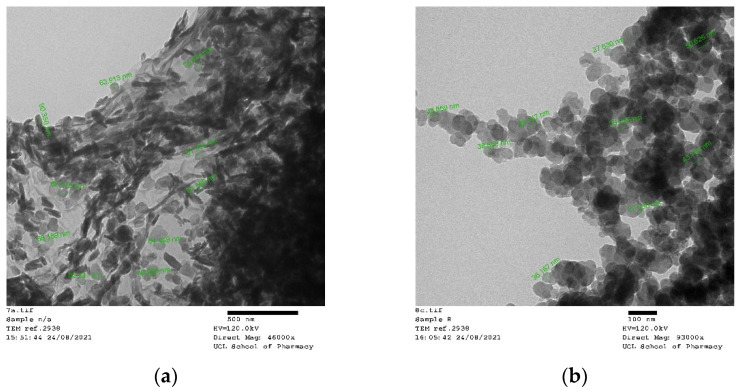
TEM images of nanoparticle (**a**) AgNP-N/A show a mixture of spherical and rod shapes with an average length of around 90 nm, and longer needle-like fibre shapes can also be observed with their length reaching over 400–500 nm; (**b**) AgNP-8 nanoparticles are mostly in spherical particle shapes sized below 100 nm.

SEM (Figure 4) and TEM (Figure 5) analyses were used to characterize nanoparticle morphology. Combining these two imaging methods can give a better understanding of the morphology of nanoparticles affected by pH. The SEM images showed that the AgNPs were abundant and overlapped each other, revealing aggregation tendency. The average particle size ranged from ~94 nm to 340 nm, with the smallest observed in AgNP-9. Figure 4f indicated a more uniformly distributed size of particle was also found in AgNP-9 samples. Meanwhile, the TEM images in the sample without pH adjustment (N/A) showed a mixture of spherical and rod shapes with a length of around 90 nm. Besides, the TEM images of AgNP-8 depicted the shape of particles as spherical with rough surfaces. 

#### 2.2.4. Antimicrobial Properties

**Figure 6:**
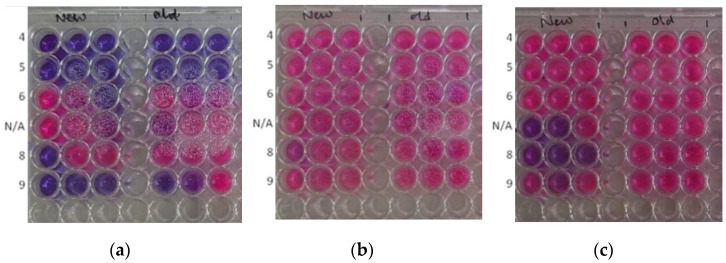
Plates after 24 h incubation based on resazurin assay with the test organism were: (**a**) *E. coli*; (**b**) *S. aureus*; (**c**) *C. albicans* [pink colour refers to microorganism growth and purple indicates the antimicrobial activity; “New”, newly synthesized nanoparticles; “Old”, nanoparticles that had been kept for ± 8 weeks; AgNP-4-9, a nanoparticle sample prepared in various pH with replicates].

**Figure 7:**
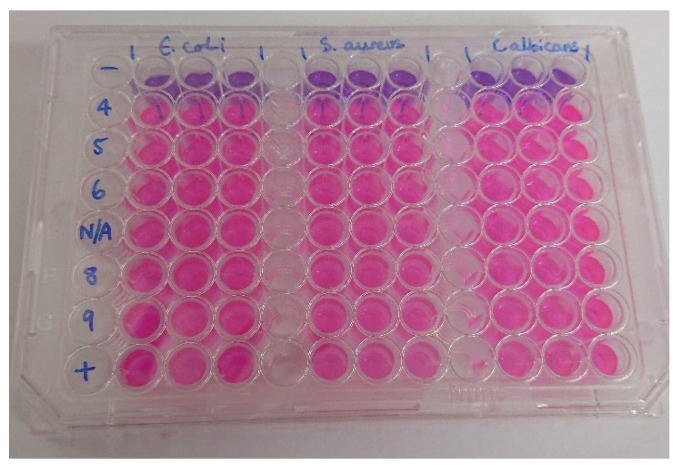
Control of plate after 24 h incubation based on resazurin assay for the test organism [pink colour refers to microorganism growth, and purple indicates the antimicrobial activity; spinach extract at pH 4–9; negative control with antibiotic gentamicin (10 ¼g/mL) for bacteria and antifungal agent clotrimazole (10 ¼g/mL) for fungi, and positive control with just microbes and broth].].

The resazurin method has been widely used to test the drug susceptibility to microorganisms, which works based on the observation of the changing colour of resazurin dye (purple) to resorufin (pink colour), indicating the presence of metabolically active cells inside the sample [34,35]. The antimicrobial test results using the resazurin method for several test organisms are provided in Figure 6 and Figure 7. The main observation here was that experimental pH influenced the resultant antimicrobial activity of AgNPs against *E. coli* and *C. albicans*. The test against *E. coli* showed antimicrobial activity on samples AgNP-4, AgNP-5, and AgNP-9, but no activity towards AgNP-6, AgNP-8 and AgNP-N/A. An additional observation was also carried out to assess the stability and effectiveness of the synthesized AgNPs by comparing the newly synthesized sample (new) with the sample that had been kept for ± 8 weeks (old). The antimicrobial activity only remained in samples 4 and 5, while sample 9 showed weak activity. The test against *C. albicans* also demonstrated weak antimicrobial activity on samples N/A and 8, which were not stable over time. The test against *S. aureus* showed no antimicrobial activity in all samples. Spinach extract at different pH had no antimicrobial activity towards all of the tested microbes.

## 3. Discussion

It is imperative to characterize the synthesized nanoparticles to achieve optimal benefit from utilizing AgNPs as antibacterial agents. The correlation between the physicochemical properties and the therapeutic benefits of nanoparticles may reveal the key parameters that are important in the drug development process. The green synthesis using *Spinacia oleracea* leaf extract that has been carried out in this study has successfully produced AgNPs in all pH conditions applied. The critical parameter in the visual investigation is the changing colour of the reaction solution from yellowish to reddish-brown, which indicates the Ag reduction in the synthesis of AgNPs [36]. The result showed that the variation in pH and synthesis time affected the colour intensity of the reaction solution. As pH increased, the colour of the reaction solution became darker. The darker colour also appeared with the increasing hours of synthesis except for samples AgNP-4 and AgNP-5. The overall UV-Vis data showed the formation of AgNPs in a relatively alkaline medium. The SPR bands were detected in the UV-Vis spectra of samples AgNP-6, AgNP-8, and AgNP-9, while there was no peak observed in samples 4, 5, and N/A. The fastest formation of AgNPs was also found in AgNP-9 samples, with the highest absorbance achieved after 3 h of synthesis. The nanoparticle sizes and shapes can be initially predicted from the characteristic of UV-Vis spectra. The single SPR band that existed in samples AgNP-6, AgNP-8, and AgNP-9 may indicate that spherical shapes had been formed [36]. Meanwhile, the red-shifting of SPR bands as the pH and time of synthesis increased often refers to smaller particles forming with more uniformly distributed particles. 

From the DLS measurement, a change in pH affects the size of AgNPs. The particle size of samples ranged from 91.75 nm to 1234 nm, with the largest found at AgNP-4 and the smallest found at AgNP-8. Samples AgNP-4 and AgNP-8 also had the widest and narrowest particle size distribution with the pDI value of 0.781 ± 0.241 and 0.213 ± 0.023, respectively. The size value obtained by the DLS technique is quite different from the value from TEM since the DLS measure the hydrodynamic radius of the particle, which includes the liquid layer around the particle. Thus, the result obtained was the estimation of t particle size with the assumption that the particle shape was spherical [29]. Basic pH generally formed AgNPs with smaller and narrower size distribution. This result is in line with the previous study’s findings [12]. The compound containing the carboxyl group tends to have a better reduction potential at basic pH from the structure-activity properties. The higher content of OH- species in the basic condition may facilitate the deprotonation of -COOH groups to -COO-, allowing the adsorption of Ag+ ions by electrostatic attraction or affinity interaction. As a result, the formation of AgNPs will most likely occur in small size and well-dispersed distribution. Nevertheless, the increasing particle size was not proportionally affected by the increasing pH since the pattern observed was fluctuating.

It was observed from the FTIR spectra that AgNPs prepared in all pH were similar and showed clear evidence of bio-reduction process and binding activity. The strong O-H stretching and C=O stretching peaks in the *Spinacia oleracea* leaf extract spectra at a wavelength around 3339 cm^−1^ and 1647 cm^−1^, respectively, were shifting to the lower wavenumber in the AgNPs spectra. This phenomenon suggests the adsorption of those functional groups on the surface of AgNPs as part of the reduction process [32]. The important phenomena that should also be pointed out are the disappearance of C-H stretching (around 2985 cm^−1^ and 2905 cm^−1^) and C-N stretching in the FTIR spectra of AgNPs, which both indicate the bioreduction by spinach constituents which transform Ag+ to Ag0 [24]. Moreover, the disappearance of C-H stretching, C-H bending, and C-O stretching peaks in AgNPs FTIR spectra may demonstrate the binding activities of those functional groups in the formation of AgNPs. 

Both morphological analyses confirmed the existence of spherical shapes in the majority of AgNPs under all pH conditions. The aggregation of particles was observed in all SEM images. Several rod shapes were observed in the TEM images of sample N/A, overlapping with the other spherical particles. Nanoparticles were known to be easily aggregated due to the high surface area, especially when the sample was kept in liquid form. 

Many factors during synthesis are known to affect the psycho-chemical properties of AgNPs, leading to the production of different behaviour and applications. Studies carried out by Manosalva et al., 2019 [37] demonstrated that pH is one of the important reaction parameters which directly affects the size distribution, agglomeration, and morphology of particles and, therefore, their antimicrobial activity. As observed in the characterization of nanoparticles in this study, the variation of pH produced AgNPs with different sizes and shapes. Furthermore, the shape variation is known to the behaviour of nanoparticles as it relates to specific endocytic cellular uptake mechanisms [20]. AgNPs were previously known to be developed as antibacterial agents due to the high ratio of surface-to-volume and crystallographic surface structure [38]. This ability is predominantly caused by the contact-killing ability as the primary bactericidal mechanism of AgNPs. 

Antimicrobial activity tests carried out in this study investigated the relationship between a certain characteristic of nanoparticles influenced by pH with its capability to inhibit the growth of certain strains of bacteria. Whilst spinach extract has been reported with antimicrobial activity at high concentrations of 60–100 mg/mL, and the spinach control extract did not show antimicrobial activity [39]. In contrast, the result showed that AgNPs exhibited antimicrobial activity against *E. coli* when prepared at AgNP-4, AgNP-5, and AgNP-9. Each type of microorganism may have a different response to the substance, depending on the easiness of the substance entering the inner cell. *E. coli*, as a Gram-negative bacteria, has a thin peptidoglycan layer separating the cytoplasmic and outer membranes. In contrast, a Gram-positive bacteria (e.g., *S. aureus*) does not have an outer membrane but a thick peptidoglycan layer. 

The antimicrobial activity of AgNPs against *E. coli* has been previously reported [40]. The study observed that the death of bacteria was initiated by the accumulation of AgNPs in the cell wall leading to the “pits” formation in the bacteria cell wall. Since the resazurin broth method allows physical contact between the microbe and the nanoparticle in a free liquid broth suspension, the antimicrobial activities shown in the sample prepared in AgNP-4, AgNP-5, and AgNP-9 indicate the higher contact produced, which may increase the probability of AgNPs accumulation in the cell wall. From the characterization of AgNPs, the average particle size of samples AgNP-4 and AgNP- 5 were known to be greater than the rest of the sample. Smaller-sized particles generally have higher antimicrobial activity as a result of their larger surface-to-volume ratio, which enables them to release a higher concentration of ions and the ability to penetrate microbial walls [14,41]. The interaction of ions and nanoparticles with the microbial cells is theorised to be one of the primary mechanisms of action of AgNPs [42]. It has been reported that Ag+ ions and AgNPs can directly interact and bind to the thiol groups of the proteins present in the microbial cell wall leading to cell wall damage, inactivation of enzymes and blockage of active sites [42,43,44]. Furthermore, AgNPs can penetrate inside microbial cells and interact with biomolecules which can lead to cell death. Morones et al. (2005) reported that smaller-sized AgNPs have higher penetration efficacy, especially towards Gram-negative bacteria [13]. 

However, the antimicrobial activity against *E. coli* in samples AgNP-4 and AgNP-5 remained after ±8 weeks of storage, while sample AgNP-9 became weaker. The better stability of antimicrobial activity produced by the larger particle formed at AgNP-4 and AgNP-5 suggests that the sizes of particles were not the only determining factor. This finding indicates that the acid pH was preferable to produce AgNPs with a certain characteristic that is effective and stable to kill *E. coli* and fungi but not effective against *S. aureus*, a typical Gram-positive bacterial pathogen. Other factors that contributed to better antimicrobial activity in AgNP-4 and AgNP-5, which were larger particles in comparison to the rest of the samples, include the surface charge and shape of the nanoparticles [45,46]. The surface charge of a nanoparticle can contribute to its antimicrobial activity through its attraction towards microbial cells. It is known that positively charged nanoparticles are electrostatically attracted to microbes which have a negatively charged cell wall. Although the surface charge of the nanoparticles was not investigated, it is possible that acid pH produced nanoparticles that had a positive (or more positive) surface charge in comparison to the alkaline pH. Gintijo et al. (2020) synthesised silver nanoparticles by altering the pH and found that the lowest tested pH produced positively charged silver nanoparticles, whilst the remaining pH resulted in negatively charged silver nanoparticles [47]. Hence, despite AgNP-4 and AgNP-5 having a large size, their surface charge may have contributed to their antimicrobial activity and led to better mechanisms of action in comparison to the rest of the samples. The shape of AgNPs in AgNP-4 and AgNP-5 that had been observed through TEM and SEM images showed that the spherical shape was responsible for producing antimicrobial activities against *E. coli*. In general, nanoparticles are assumed as spherical, which in reality, may exist in an irregular shape with a variety of geometric features [48]. The spherical form was also previously reported to produce antibacterial activity against Gram-positive and Gram-negative bacteria strain lines [49], putting them as a good candidate for replacing the use of antibiotics. 

In the antibacterial test using *S. aureus*, no antibacterial activity was found in all samples. It was assumed that the synthesized AgNPs, under all pH conditions, could not penetrate the thick peptidoglycan wall of Gram-positive bacteria, causing the absence of antibacterial activity. Whilst AgNPs have been reported to have antimicrobial activity against certain Gram-positive bacteria, studies have shown that Gram-negative bacteria are more susceptible towards AgNPs in comparison to Gram-positive bacteria. The thick peptidoglycan wall provides a stronger barrier which can decrease the diffusion of AgNPs and Ag+ ions into the cell and therefore prevent cytoplasmic damage [50,51,52,53]. This also suggests that the uptake of AgNPs and Ag+ ions is required for effective antimicrobial activity.

Meanwhile, a weak antimicrobial activity was observed against *C. albicans* in samples N/A and 8. The weak activity indicated the ability of AgNPs to inhibit some organisms in the well but not enough to kill them all. The antifungal activity of AgNPs against Candida species has been previously investigated in several studies using the smaller nanoparticles [54,55] and showed the formation of inhibition zones with various concentrations of AgNPs [56]. The DLS measurement showed that the particle size of samples AgNP-N/A and AgNP-8 were relatively small compared to the other sample due to the basic pH condition. Theoretically, the higher surface volume ratio in a smaller size of particles will increase the adhesion of particles to the surface of the microorganism cell wall, which may increase particle infusion or diffusion processes to enter the cell walls. The smaller size nanoparticle with a larger surface area will improve the antimicrobial activity and increase its chemical stability [57]. Based on the TEM and SEM images, the particles formed in the N/A were a mixture of rod and spherical shape, while sample AgNP-8 contained spherical particles with rougher surfaces in general. However, the antimicrobial activity against *C. albicans* was not stable over time. This changing property may be attributed to the aggregation of nanoparticles that easily occurs due to the high surface area, which provides a high probability of interaction between particles. This result indicated the effect of pH to induce the antimicrobial activity of the nanoparticles, such as AgNPs, towards certain types of bacteria and influence its stability and effectiveness. Most of the previous publications demonstrated that the particles are effectively antimicrobial normally in their sizes around 10–70 nm [58,59], which were much smaller in comparison with this work that synthesized with a size range over 90–100 nm. However, the nanoparticles in this work are in the range of 90–1000 nm overall. And the dynamic range of the nanoparticles is widely spread with an adjustment of synthesis pH, which could be advantages in speculations based on the test results in Figure 4 and Figure 5, shows that a wide range of Gram-negative bacteria and fungi pathogen could be inhibited with a low cost of an environmentally friendly production route developed by this project.

## 4. Materials and Methods

### 4.1. Materials

#### Materials and Chemicals

*Spinacia oleracea* leaves were identified and collected from a local grocery store in London, United Kingdom. Ethanol, Silver Nitrate (AgNO_3_), Hydrochloric Acid (HCl), and Sodium Hydroxide (NaOH) were purchased from Sigma Aldrich, UK. All chemicals were analytical grade and used without further purification.

### 4.2. Spinacia oleracea Leaf Extraction

#### 4.2.1. Maceration of *Spinacia oleracea* Leaf

Ten grams of *Spinacia oleracea* leaves were weighed and rinsed using tap water, followed by distilled water twice for each. The leaves were cut into pieces, soaked in the 100 mL mixture of ethanol and distilled water (1:1), and left overnight. The *Spinacia oleracea* leaf macerate was filtered and centrifuged for 10 min at 7000 rpm. The supernatant was filtered using Whatman filter grade #1, and the *Spinacia oleracea* leaf extract was obtained.

#### 4.2.2. Green Synthesis of AgNPs Using *Spinacia oleracea* Leaf Extract

AgNO_3_ will dissociate according to the following reaction:

(1) $$AgNO_{3}\to Ag^{+}+NO_{3}^{-}$$

Visual observation of Ag reduction in green synthesis is seen by a colour change to yellow-brown at different levels of intensity [36]. In this study, the plant-mediated green synthesis of AgNPs was adapted from [24], which used red spinach (*Amaranthus Tricolor* L.) leaf extract. In brief, AgNPs were synthesized using 10% *v*/*v* of *Spinacia oleracea* leaf extract (5 mL) and 45 mL of 1 mM AgNO_3_ solution. The experimental pH was adjusted using HCl 0.1 M or NaOH 0.1 M to pH 4, 5, 6, 8, and 9, respectively. These samples are referred to as AgNP-4, AgNP-5, AgNP-6, AgNP-8 and AgNP-9, respectively. The experimental pH without alternation was observed to be pH 6.5, therefore this sample was used without any pH adjustment to compare the properties of the altered pH samples to the unaltered. This sample is referred to as AgNP-N/A. 

A control was prepared using the same procedure without the addition of *Spinacia oleracea* leaf extract. The synthesis was conducted over 3 h at 70 °C and continuous stirring at 300 rpm. This was followed by a purification step to separate AgNPs from the debris that existed, with the following steps: 1 mL of sample was centrifuged for 15 min at 15,000 rpm, and the supernatant formed at the top of the mixture was discarded and replaced with new distilled water. The purification step was carried out twice.

### 4.3. Sample Characterization

#### 4.3.1. Visual Observation

The samples were placed against a white background and observed for any changing colour at the start (0 h), after 2 h, and after 3 h of synthesis.

#### 4.3.2. UV-Visible Spectroscopy

UV-Vis spectroscopy characterization was carried out using Lambda 2 UV/VIS Spectrometer (Perkin Elmer) connected with UV Win-Lab as the software between 300–500 nm to detect the Surface Plasmon Resonance (SPR) Band between 390–450 nm, which confirms the presence of Ag^−^ [25]. Characterisation at the beginning (0 h), after 2 h, and after 3 h of synthesis was performed.

### 4.4. Nanoparticle Characterization

The size, shape and morphology of green synthesised AgNPs were conducted using dynamic light scattering (DLS), scanning electron microscopy (SEM) and transmission electron microscopy (TEM). Antimicrobial properties at time 0 and after 8 weeks were evaluated using the Resazurin method. 

#### 4.4.1. Dynamic Light Scattering

The particle size and polydispersity index (pDI) were measured using the Zetasizer Nano series (Malvern) with a DLS system connected with Zetasizer Software.

#### 4.4.2. Fourier Transform Infrared Spectroscopy

FTIR analysis was carried out by placing a drop of sample on the ATR surface of Frontier FTIR Spectrometer (Perkin Elmer), which connected with Spectrum 10 as the software. The instrument is set to accumulate spectra in the IR region of 4000 cm^−1^ to 650 cm^−1^.

#### 4.4.3. Scanning Electron Microscopy

A droplet of the sample was attached to a self-adhesive carbon disc mounted on a 25 mm aluminium stub. The stub was coated with 10 nm of gold using a sputter coater. The stub was then placed into an FEI Quanta 200 FEG SEM (Netherlands) for imaging at 10 kV accelerating voltage using secondary electron detection.

#### 4.4.4. Transmission Electron Microscopy

Liquid samples for TEM were dropped with a Pasteur pipette onto a carbon/formvar coated copper grid. After 15 s, the excess sample was blotted off with filter paper. Then a drop of stain was added if required and blotted after 15 s. The grid was placed into a specimen holder and inserted into a Phillips/FEI CM 120 BioTwin TEM (Netherlands) for imaging at 120 kV.

#### 4.4.5. Bacteria, Fungi Preparation and Antimicrobial Analysis

*E. coli*, *S. aureus*, and *C. albicans* (ATCC 2091) were kindly provided by The University of Hertfordshire microorganism collection and grown in a shaking incubator (200 rpm at 37 °C) for 24 h in broth (Nutrient broth for bacteria, yeast peptone dextrose broth for fungi), respectively. Microbes were diluted to ~3 × 10^7^ CFU in Mueller Hinton broth using CE 1021 spectrophotometer (Cecil, UK) at 600 nm. Nanoparticles were vortexed (10 s), placed in the sonic bath for 1 min, vortexed again, and placed in the sonic bath for a further 1 min. In a 96-well plate, 100 μL of microbes were treated with 100 μL of nanoparticles suspension. Controls were also added for each of the microbes; antibiotic gentamicin ( at a concentration of 10 ¼g/mL) and antifungal agent clotrimazole (at a concentration of 10 ¼g/mL) were used as a control for the bacteria and fungi, respectively, and microbes without nanoparticle treatment as a positive control. Additionally, 100 ¼l of the spinach extract was tested for antimicrobial effects. Plates were incubated at 37 °C for 24 h. A 25 μL of resazurin dye (0.02%) was added to each well and incubated again for 24 h. Colour change was observed and recorded. All broth, resazurin, gentamicin and clotrimazole were purchased from Sigma-Aldrich, London, UK.

## 5. Conclusions

To conclude, pH-dependent green synthesis of AgNPs using *Spinacia oleracea* leaf extract was successfully conducted; the sizes and shape of the particles were greatly determined by the pH of synthesis, which ultimately affects the production of antimicrobial activity against *E. coli* and *C. albicans*. Changing AgNPs’ shape and size by the variation of pH synthesis causes the different accumulation of AgNPs within the microbe’s cell wall, which mainly determines the death of the microbe. Antimicrobial activity against *E. coli* and *C. albicans* of AgNPs synthesized in alkaline conditions was found to be reduced over time. Meanwhile, the change in pH did not affect the antimicrobial properties of AgNPs against *S. aureus.* Therefore, it is necessary to find the optimum size and shape of AgNPs by modifying some of the parameters, including the pH of synthesis. This research contributes to the development of AgNPs as a new alternative in dealing with infectious diseases. By knowing the critical parameters that need to be controlled in the synthesis process, the AgNPs can be designed with optimal characteristics that will effectively kill microorganisms, especially those that have been resistant to antimicrobials. 

## Figures and Tables

**Table 1:** *λ*max and absorbance of the SPR band of samples AgNP-6, AgNP-8, and AgNP-9 after 3 h.

pH	*λ*max	Absorbance
AgNP-6	418–419 nm	0.0326
AgNP-8	419 nm	0.1244
AgNP-9	422–424 nm	0.2845

**Table 2:** Particle size and pDI value of the sample prepared in various pH.

Parameter	Samples					
	AgNP-4	AgNP-5	AgNP-6	AgNP-N/A	AgNP-8	AgNP-9
Z-average	1234 d.nm ± 663.30	193.9 d.nm ± 30.98	184.2 d.nm ± 18.55	405.3 d.nm ± 161.6	91.75 d.nm ± 8.31	263.6 d.nm ± 42.27
pDI	0.781 ± 0.241	0.494 ± 0.234	0.249 ± 0.029	0.418 ± 0.180	0.213 ± 0.023	0.332 ± 0.074

## Data Availability

Data is available on request.
